# Research on Monitoring of Gymnastics Facilities and Intelligent Optimal Distribution of Gymnastics Venues Based on Internet of Things

**DOI:** 10.1155/2022/6164448

**Published:** 2022-09-09

**Authors:** Hongbo Liu, Yuzhen Wang

**Affiliations:** School of Physical Education, Henan Polytechnic University, Henan, Jiaozuo 454003, China

## Abstract

In view of the low level of gymnastics facilities monitoring and intelligent management of gymnastics venues, which cannot effectively manage gymnastics venues in real time, this paper proposes a method of gymnastics facilities monitoring and intelligent optimization distribution of gymnastics venues in the Internet of Things. This paper will build an information monitoring model, introduce a particle swarm optimization algorithm to participate in the location layout, and explore the actual effect of gymnastics and the Internet of Things. The research results show that (1) the system can measure the mechanical error of facilities and ensure that the controllable fault tolerance rate is less than 1%. (2) The quality of system monitoring is evaluated in accuracy, time, delay, and satisfaction, and the results are basically satisfactory, but the accuracy and time still need to be improved. (3) Using the evaluation system of sports facilities to test the temperature, humidity, facility pressure, and energy consumption suitable for gymnastics and to verify the injury tendency of athletes. When the damage tendency is between 70% and 100%, the actual damage rate is 1. (4) The speed of the PSO algorithm is faster than other methods, which is used to optimize the layout of gymnastics venues and has a certain role in promoting the construction of gymnastics venues. The system model designed in this paper performs well in gymnastics and needs to be further improved and optimized.

## 1. Introduction

The Internet of Things era breaks down the barrier between reality and virtuality and launches a great change in intelligent and efficient life. Under the background of the 5G network, all walks of life have entered a new stage of development by applying the Internet of Things, and at the same time, massive data information has been formed, and great progress has been made in connecting everything in the world. From the aspect of sports events and industry development, with the Internet of Things changing life, people's spiritual needs have become rich and diverse, and the attention to sports culture has increased. How to innovate and develop sports on the existing technology has become a new research topic. Gymnastics, as a sport with both aesthetics and mechanics, has high requirements for technology, venues, and facilities in order to avoid injuries to athletes as much as possible. This paper will face the gymnastics, to build an intelligent Internet of Things system, which can be used for both teaching and scientific research and can also be used for sports events. The system model meets the monitoring requirements of gymnastics facilities and can also be connected with sports facilities through intelligent wearable devices to measure the physical condition of athletes. In addition, we should optimize the location and layout of gymnastics venues and strive for the most intelligent distribution layout.

Because the research on sports in China is still in the preliminary theoretical research stage, there are few references in the application field of the Internet of Things, and the experimental data are missing or incomplete. Therefore, in this case, we read a large number of experiments and cases using modern information technology for reference, so as to build the ideological line of the paper, and hope to fill in the blank of this research by the content of this article. We use IoT to perceive sports resources for development and utilization and build the application architecture of related content [1]. Using the advantages of data mining and IoT, an intelligent sports health management system is constructed to control physical fitness [2]. H2T Internet of Things is used to monitor heart rate data, and NFC and mobile intelligent devices are used to build a sports evaluation platform [3]. This paper studies the overall structure and system functionality of an intelligent venue system and optimizes the design based on IoT [4]. We optimize various methods and technical routes, integrate them with a physical fitness test, and transform the original equipment and systems [5]. We organize the sensors and application principles of the Internet of Things and apply wearable devices to sports science [6]. This paper expounds the scope and main contents of sports facilities detection and puts forward prospects and suggestions for sports function detection [7]. Taking the public sports facilities of national fitness in Hebei Province as the object, this paper studies the resource allocation and service optimization [8]. We design a model to monitor the emission law of volatile pollutants in large stadiums and gymnasiums [9]. Combined with the concept of low carbon, we plan the resettlement, construction, and service quality of urban community sports facilities [10]. According to the experience and enlightenment of Finnish schools, referring to the result-oriented concept of sports quality monitoring, this paper studies the monitoring framework structure and system operation mechanism [11]. The intelligent management and monitoring system of sports training hall based on the Internet of Things is deeply analyzed and studied [12]. Based on automatic detection technology, sports machinery is detected and processed, and errors are analyzed [13]. Discrete wavelet transform (DWT) and random forest algorithm are used to construct sports injury training set, and the monitoring system of athletes' injury possibility is established [14]. Drawing lessons from the American experience, this paper analyzes the regional distribution characteristics and causes of venues in the four major sports leagues and provides theoretical and practical guidance for optimizing the layout [15]. The Internet of Things proposed in this paper is applied to the monitoring and management of gymnastics facilities, which can effectively improve the intelligence and utilization rate of sports venues. Compared with the application of other Internet of Things scenarios, it lacks the overall design and optimization. There are few application scenarios in sports venues, especially gymnastics venues, and the intelligent effect is not realized from the theoretical point of view and combined with artificial intelligence technology.

Applying Internet of Things technology in gymnastics venues can effectively improve the economic efficiency of venues. In this paper, we consider evaluating the quality of system monitoring in gymnastics venues in terms of accuracy, time, delay, and satisfaction, and the results are basically satisfactory, but the accuracy and time still need to be improved. Using sports facilities evaluation system, the temperature, humidity, facility pressure, and energy consumption suitable for gymnastics are tested to verify athletes' injury tendencies.

## 2. Theoretical Basis

### 2.1. Concept of Internet of Things Technology

International Organization for Standardization [16]: The technical term of this technology can be interpreted as “Internet of Things.” It first appeared in AUTON-ER Research Center in 1999, which can realize intelligent identification and management and combine sensing devices with the Internet. Nowadays, IoT goes beyond the original traditional network and eliminates the communication restrictions between people. It belongs to a form of information sensing and network integration. It has broad prospects and is widely used in the interactive scenes of “IoT” in all walks of life, showing brand-new vigor and vitality. It uses sensing devices to connect to a network to promote information communication and data exchange between people and things, things and things; with the help of various computer technologies, the requirements of monitoring, identification, intelligent management, and positioning can be perfectly realized. It is worth noting that the Internet of Things industry is a new strategic industry, which still has great potential and room for transformation. The technical standards and commercial development related to it in China are still in the primary stage of development. People's concept of IoT is still vague, and the relatively sophisticated core depends on the introduction of foreign technology, so more efforts should be made in this respect.

**Figure 1:**
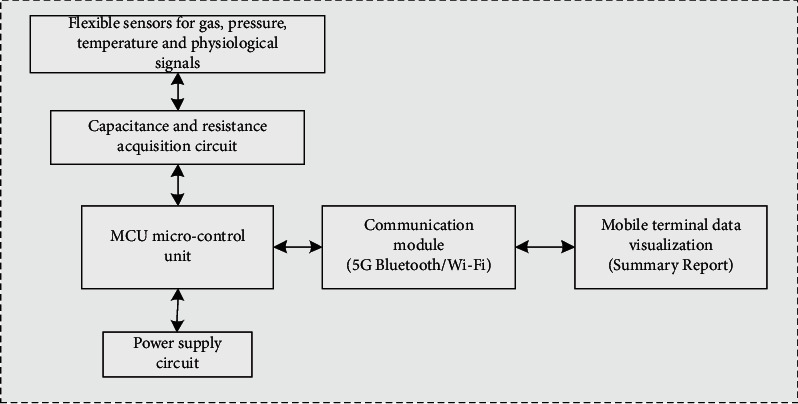
Display of sensor data monitoring structure.

Transducer/Sensor [17]: That is, a device can be used for detection. It can change the sensed measurement information into the required information by law and meet the requirements of data collection, storage, and recording. In the special network of IoT, the basic equipment plays a vital role in various sensors. Without sensors to realize automatic detection and control functions, objects cannot be anthropomorphic and have senses such as touch, taste, and vision, and human beings will lose a channel to obtain accurate and reliable information. In this paper, we will install different sensors in gymnastics facilities to detect pressure, temperature, gas, and other aspects in order to create a good suitable sports environment. Then, wearable intelligent sensors are installed on the athletes, which work together with the sensors on the facilities to monitor the physiological signals of the athletes and intelligently analyze the injury possibility of the athletes doing gymnastics based on environmental factors. The sensor hardware architecture realized in this paper can receive the sensor data sent by the microcontrol unit through communication technology and finally present a visual interface for easy viewing as shown in Figure 1.

### 2.2. DWT and Random Forest Algorithm

DWT [18]: It is called discrete wavelet transform. It can discretize the scale parameters and displacement parameters of the basic wavelet. According to the three-dimensional data collected by sensors, the acceleration data including *X*, *Y*, and *Z* directions are processed, and the characteristics can be processed and summarized by the computer after transformation. Each wavelet is scaled by translation, and the acceleration is replaced by a binary discrete wavelet. Formula (1) sets the total energy release in a period of time as *E*_1_ and decomposes it into *i* levels, where *A*_*i*_ represents the approximate coefficient of wavelet; *A*_*i*_^1^ represents the result after transposition; *D*_*i*_ represents the actual coefficients of the wavelet. Formula (2) represents the energy ratio of approximate coefficients; formula (3) represents the energy ratio of coefficients in actual cases.

(1) $$\begin{array}{c} E_{T}=A_{i}A_{i}^{T}+\overset{i}{\underset{j=1}{\sum }}D_{j}D_{j}^{T}, \end{array}$$

(2) $$\begin{array}{c} EDR_{A}=\frac{A_{i}A_{i}^{T}}{E_{T}}, \end{array}$$

(3) $$\begin{array}{c} EDR_{D_{j}}=\frac{D_{i}D_{i}^{T}}{ET},j=1,...,i. \end{array}$$

This paper introduces how DWT transforms the original three-dimensional acceleration data into binary processable data. We also need to introduce classifiers in machine learning in order to automatically classify the activities of athletes efficiently and accurately so as to effectively monitor the sports situation of athletes and improve the success rate of exploring the possibility of injury. In this paper, the stochastic forest fusion algorithm with the statistical self-sampling method as the ideological root is adopted. A strong predictor [19] is composed of a plurality of weak decision-makers [20]. It has a good performance in practical application. The basic steps of the algorithm are as follows:

(1) Bootstrap resampling is carried out on the original data to generate training sets, and then, a decision tree is formed
  (4) $$\begin{array}{c} T\left(\theta \right):\left\{T\left(x,\theta_{1}\right)\right\},\left\{T\left(x,\theta_{2}\right)\right\},...,\left\{T\left(x,\theta_{k}\right)\right\}. \end{array}$$
(2) Achieve maximum prediction [21]
(3) The observed value *X* belongs to leaf nodes and is not equal to zero, so the distribution weight is defined
  (5) $$\begin{array}{c} \omega_{i}\left(x,\theta \right)=\frac{1\left\{X_{i}\in R_{l\left(x,\theta \right)}\right\}}{\left\{j:X_{i}\in R_{l\left(x,\theta \right)}\right\}},i=1,2,...,n. \end{array}$$
(4) Set the sum of the weight values of a single decision tree as 1, and weigh the observed values of each dependent variable
  (6) $$\begin{array}{c} \hat{\mu }\left(x\right)=\overset{n}{\underset{i=1}{\sum }}\omega_{i}\left(x,\theta \right)Y_{i}. \end{array}$$
(5) The predicted values shown in formula (6) are summed up, and the weights of formula (5) are combined to finally obtain the weights of each observed value
  (7) $$\begin{array}{c} \omega_{i}\left(x\right)=\frac{1}{k}\overset{k}{\underset{i=1}{\sum }}\omega_{i}\left(x,\theta_{i}\right)Y. \end{array}$$

Finally, the predicted value is obtained.

(8) $$\begin{array}{c} \hat{\mu }\left(x\right)=\overset{n}{\underset{i=1}{\sum }}\omega_{i}\left(x\right)Y_{i}. \end{array}$$

### 2.3. Facility Site Selection and Site Layout

#### 2.3.1. Address Selection of Site

The construction of gymnastics facilities belongs to the government construction project. Therefore, before formally optimizing the internal layout of the venue, we need to analyze the principle of venue location. Considering the principles of fairness, efficiency, integration, and networking, we should not only ensure the transportation convenience, benefits, facilities, and services of venues but also arrange facilities from the overall situation. Two classical models, P-center [22] and *P*-median [23], are selected.

*(1) Classical P-Center Model*. This model can embody the fairness principle of venue location and can find the maximum and minimum distance from any demand point to the location. Let *I* be the number of demand points, where *i*={1,2,3..., *I*}; *J* is the number of candidate position points, where *j*={1,2,3..., *J*}; *P* is the number of newly added sites; *d*_*ij*_ is the distance between *i* and *j*. Get the mathematical expression of the model:

(9) $$\begin{array}{c} F_{1}=\mathrm{Min}\left\{\mathrm{Max}\underset{j\in J}{\sum }d_{ij}y_{ij}\right\}\;\forall i, \end{array}$$

(10) $$\begin{array}{c} st.\underset{j\in J}{\sum }y_{ij}=1,\;\forall i, \end{array}$$

(11) $$\begin{array}{c} y_{ij}\leq X_{j},\;\forall i,j, \end{array}$$

(12) $$\begin{array}{c} \underset{j\in J}{\sum }X_{j}=P, \end{array}$$

(13) $$\begin{array}{c} X_{j}\in \left\{0,1\right\},\;\forall j, \end{array}$$

(14) $$\begin{array}{c} y_{ij}\in \left\{0,1\right\},\;\forall i,j. \end{array}$$

Formula (9) refers to minimizing the length between the location points closest to the demand position. Formula (10) assigns each demand point to its nearest location. Formula (11) ensures that each candidate site is equipped with a facility site. Formula (12) indicates that there are P places in the candidate sites. Formulas (13) and (14) indicate binary constraints on variables.

*(2) Classical P-Median Model*. This model was first proposed in 1964 [24]. It mainly reflects the efficiency principle of site selection and calculates the shortest time from each demand point to the selected address. The mathematical expression of the model is obtained. Except for different objective functions, the constraint conditions of the model are the same as those of the *P-*center model, so it is not listed separately here.

(15) $$\begin{array}{c} F_{2}=\mathrm{Min}\underset{j\in I}{\sum }\underset{j\in J}{\sum }d_{ij}y_{ij},\;\forall i,j. \end{array}$$

#### 2.3.2. Intelligent Optimal Distribution of Site

Determining the position of objects [25] is the basis of all layout optimization problems. In this paper, the distribution of sports facilities in gymnastics venues can be simplified as a continuous space layout optimization problem. Taking a venue as an example, the length and width of the venue are *H* and *K*, respectively, so it is necessary to set up *D* sports facilities.

(16) $$\begin{array}{c} F=\left(f_{1},f_{2},...,f_{D}\right). \end{array}$$

Set the total cost of moving sports facilities in the venue and make mathematical modeling. Solve the minimized objective function:

(17) $$\begin{array}{c} \min\;C=\overset{S}{\underset{s=1}{\sum }}\overset{D}{\underset{j=1}{\sum }}\overset{D}{\underset{k=1}{\sum }}P^{S}\cdot Q_{jk}^{S}\cdot D_{jk},\\ D_{jk}=\left|x_{j}-x_{k}\right|+\left|y_{j}-y_{k}\right|\\ A_{jk}\cdot B_{jk}=0. \end{array}$$

## 3. Research on the Whole Model Framework

### 3.1. Overall Functional Design

**Figure 2:**
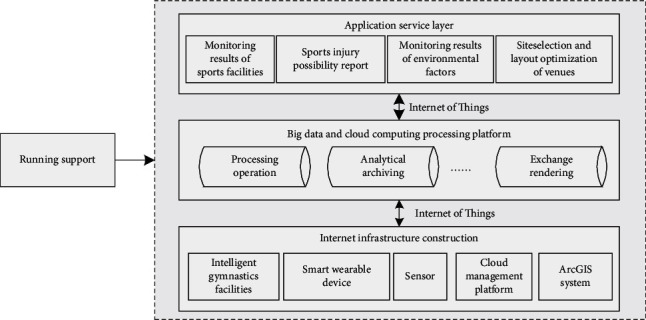
Overall architecture of monitoring and optimization facility system.

In order to realize the construction of gymnastics venues and facilities, it is necessary to build a system engineering that can support the operation. The system is mainly used to monitor the environmental data of facilities, select the best venue address, and intelligently optimize the venue's spatial layout. The whole system is connected through the Internet of Things, and the system facilities are constructed through intelligent facilities, sensors, intelligent wearable devices, cloud management platform, and ArcGIS system. After completing a series of Internet infrastructures, the system automatically uploads the data of daily movement process to the cloud by using an automatic classification method and can quickly extract, cloud, classify, and exchange data through big data and cloud computing platform. Finally, we can view and use the results through the visual operation interface of the system. The specific design situation is shown in Figure 2.

### 3.2. Mechanical Error Diagnosis of Gymnastics Facilities

Our facilities and equipment for gymnastics are mainly electronic sports machinery. Due to the limitation of technical problems, it is difficult to control the quality of these machines in actual production and operation, so it is easy to cause inaccurate measurement results due to mechanical errors. In this section, we use some measures to detect and analyze the errors of sports facilities and strictly control the product quality of sports facilities. In order to achieve this step, we take the internal hardware circuit as the main research goal and extract error features. Where *R* is composed of different relative leakage resistors; *C* is the parallel equivalent of different relative ground capacitors. *ω* stands for frequency, and *φ* stands for the phase difference angle between error current and *U*_0_; we can use tan *φ* to represent the mechanical error characteristics, as shown in the following formula :

(18) $$\begin{array}{c} \left\{\begin{array}{c} R=\frac{1}{3/r_{0}+1/r_{L}},\\ C=3C_{0}, \end{array}\right.\\ I_{\delta }=\frac{U_{0}}{R}+j\omega C_{0}+\frac{U_{0}}{j\omega L}=I_{R}+j\left(I_{C}-I_{L}\right),\\ \tan\;\phi =\frac{\left(I_{C}-I_{L}\right)I_{R}}{I_{C}I_{\delta }}RC. \end{array}$$

### 3.3. Damage Likelihood Monitoring

Based on DWT and random forest algorithm, a large-scale sports injury monitoring database is constructed by fusing the data collected by wearable smart devices and facility sensors. According to the motion monitoring results, various effective motion displacement curves are drawn, and the mechanical feedback of sports injuries is analyzed. Phase shift registration method is adopted.

(19) $$\begin{array}{c} x_{i}^{*}\left(t\right)=x_{i}\left(t+\delta_{i}\right),\\ SSE=\overset{N}{\underset{i=1}{\sum }}\int {\left[x_{i}\left(t+\delta_{i}\right)-\mu \left(t\right)\right]}^{2}ds. \end{array}$$

Read the waveform until it does not change significantly, and get the activity classification. Look at the curve trend of eigenvalue and motion displacement, and discuss whether there is the possibility of injury.

(20) $$\begin{array}{c} SSE_{n-1}\leq SSE_{n}\approx SSE_{n+1}. \end{array}$$

### 3.4. Location and Layout Optimization

Combining *P*-median and *P*-center models, considering the original gymnastics facilities, the location function is modified.

(21) $$\begin{array}{c} W=\alpha_{1}F_{1}+\left(1-\alpha_{1}\right)F_{2}. \end{array}$$

Gymnastics field layout problem needs to be in a certain internal space, and all the equipment and all kinds of resources are needed for gymnastics reasonable organization and layout. Venues need to determine the venues that meet all needs, and set up competition venues, warm-up venues, training venues, and competition platforms according to the level, scale, and athletes of competition or training. In order to ensure that athletes have good space and visual effects, there should be no direct natural light, and the safe distance and space between edges should be guaranteed. Optimizing the layout of gymnastics venues with PSO intelligence is as follows:

(22) $$\begin{array}{c} \begin{array}{c} P=\left(x_{1},...,x_{d},y_{1},...,y_{d}\right),\\ V=\left(v_{x1},...,v_{x\;d},v_{y1},...,v_{y\;d}\right),\\ F=\frac{1}{\overset{S}{\underset{s=1}{\sum }}\overset{D}{\underset{j=1}{\sum }}\overset{D}{\underset{k=1}{\sum }}p^{s}\cdot Q_{jk}^{s}\cdot D_{jk}+Pe\left(i\right),}\\ Pe\left(i\right)=1/2\left(t_{1}\cdot \left(\left|h\left(i\right)\right|+h\left(i\right)\right)+t_{2}\cdot \overset{4}{\underset{b=1}{\sum }}\left(\left|g_{b}\left(i\right)\right|+g_{b}\left(i\right)\right)\right), \end{array}\\ \omega =\omega_{\max}-\frac{\omega_{\max}-\omega_{\min}}{k_{\max}}\cdot k. \end{array}$$

## 4. Experimental Analysis

### 4.1. Introduction of Experimental Environment

In this section, we show the software or hardware used in the experimental part as shown in Table 1.

### 4.2. System Monitoring Test

#### 4.2.1. Mechanical Error Evaluation of Facilities

**Figure 3:**
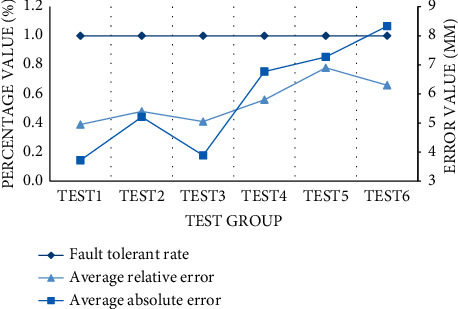
Monitoring results of facility accuracy error.

For all sports equipment, it is impossible to achieve 100% error-free production, and it can be qualified production within an error range. Especially for sports equipment, when the precise value is emphasized, the analysis of electro-mechanical error is also very important for the normal operation of the equipment. In this paper, the mechanical error of our control facilities is less than 1% (i.e., fault tolerance rate), and if it exceeds this value, it will be deemed as unqualified. We set up six test groups and got the average relative error value of each group, the unit is millimeter, and the results are not more than 9 mm. Looking at the curve trend in the graph, we can find that the average relative error curve of facilities is below the fault tolerance curve. This means that the average relative error of all test groups is below 1%, which is within the qualified error range. Among them, the fifth test group has the highest error, which can reach 0.78%. The first test group has the lowest error, only 0.39%, as shown in Figure 3.

#### 4.2.2. Monitoring Quality Evaluation

**Figure 4:**
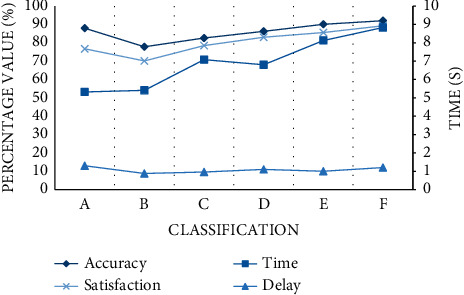
Overview of system quality.

In this section, the main test system monitors the overall quality of the facility, for example, the accuracy of facility test results, the duration, and delay of use. Six kinds of gymnastics facilities that can be monitored are selected, numbered A∼F. According to the curve trend in the figure, we can find that the monitoring time of the system is between 5 s and 10 s, and the overall time delay is less than 1.5 s. In terms of accuracy, the results of facilities E and F are all over 90%. Except for facility B, the accuracy is 77.80%, and the test results of other facilities A, C, and D are all between 80% and 90%. In addition, in order to evaluate and monitor sports facilities more professionally and comprehensively, three experts and scholars in related sports fields were invited to conduct a systematic satisfaction evaluation. It can be found that the satisfactory results are between 70% and 90%, and the overall effect is good. However, the monitoring results of A, B, and C facilities are 76.70%, 70.10%, and 78.45%, respectively. Experts think that these three gymnastics facilities still need to be improved as shown in Figure 4.

#### 4.2.3. Evaluation of Monitoring Results

Usually, the temperature of gymnastics training venues is 22–23 degrees, and the humidity index is 22–38. Generally, it can be fine-tuned according to the actual weather conditions of the day. It is necessary to keep the indoor air of the site fresh and free from harmful gases or special gases causing human discomfort. We monitored the sports facilities of gymnastics venues 1 to 5 and summarized the following results as shown in Table 2.

**Figure 5:**
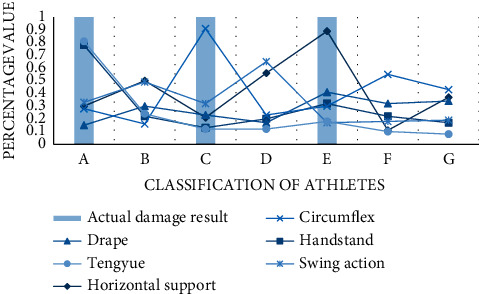
Damage monitoring.

Subsequently, through the monitoring of wearable intelligent devices, this paper counts the sports injury data of athletes under different gymnastics movements. Need to declare one point is because the gymnastics movement selected in this experiment belongs to more professional and difficult items, the data will be biased, resulting in a higher injury tendency. Let 7 athletes numbered A to G perform 6 gymnastics exercises on sports facilities. The controlled room temperature is 22 degrees, the air wettability is 35 degrees, the gas quality is good, the facility pressure detection and physiological signal detection devices can operate normally, and the energy consumption is in a general state. After testing, the system analyzes the possibility of damage according to the detected data. According to the curve, we find that the actual injury rate of athletes with an injury tendency between 70% and 100% is 100%, which proves the accuracy and effectiveness of monitoring and predicting effects as shown in Figure 5.

### 4.3. Results and Evaluation of Intelligent Layout Optimization

#### 4.3.1. General Situation of Venue Facilities Selection Location

**Figure 6:**
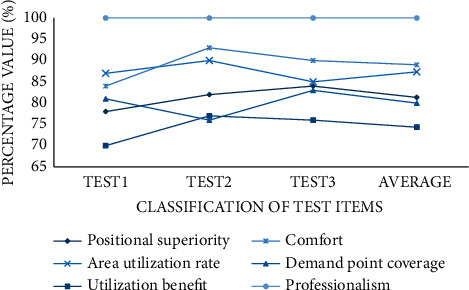
Rationality score for site layout optimization.

After selecting the appropriate gymnastics site, the system optimizes the distribution of the facilities intelligently. In this section, the final results are evaluated for three rounds. Score the site location evaluation, overall use efficiency, coverage demand points, layout cost, area utilization rate, and layout comfort and professional qualification. We can find that the location of the venue chosen in this paper is convenient and superior and can cover more demand points, and the use benefit can reach 74%. After optimizing the layout of facilities in the site, the distribution of facilities is more reasonable, with a professionalism of 100%, and both the area utilization rate and comfort are higher than 85% as shown in Figure 6.

#### 4.3.2. Comparison of PSO Optimization Algorithms

**Figure 7:**
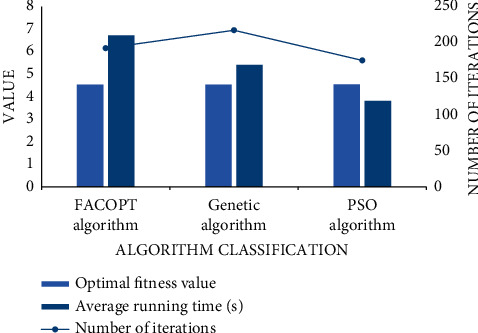
Comparison of different algorithms.

In this paper, according to the global optimization, the PSO algorithm is used to plan and integrate all the internal resources or equipment in a given limited stadium to solve the problem of site distribution. We add the FACOPT algorithm and genetic algorithm to compare the layout results. It can be found that the proposed method can obtain the optimal fitness value under the lower iteration times, and the shortest time is about 3.83 s as shown in Figure 7.

#### 4.3.3. Optimization Results of Continuous Spatial Distribution

**Figure 8:**
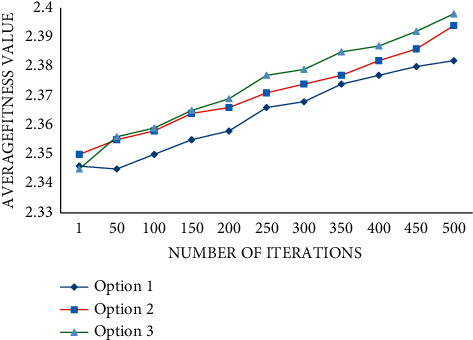
PSO optimization process of different schemes.

When optimizing the space of the site, there are usually many design schemes. In order to improve the credibility and reliability of the results, we will go through 500 iterations of different schemes and observe their average fitness values. According to the curve in the figure, we can know that with the increase in iteration times, the average fitness values of the three schemes also increase. Among them, the effect and advantage of scheme 3 are the most obvious as shown in Figure 8.

## 5. Conclusion

In this paper, flexible pressure, temperature, physiological signals, and other sensors are used to intelligently collect sports equipment data; wavelet transform and random forest algorithm are used to intelligently monitor gymnastics activities; ArcGIS system and particle swarm algorithm are used to optimize the layout of facilities. The final experimental results show that the application of GIS technology promotes the results of site selection and layout, and the model solution is efficient. Through the sensor data monitoring system, the equipment, environment, and athletes can be detected in real time. The Internet of Things can better improve the management efficiency of facilities and venues for China's sports industry and help to improve the overall level of competition. Although this paper has obtained good experimental data and research results but is affected by various factors and conditions, the research on this subject should be refined, further adjust the system environment and work, and put forward more stringent new requirements. The location of the venue selected in this paper is convenient and superior, which can cover more demand points, and the use efficiency can reach 74%. After optimizing the layout of facilities in the site, the distribution of facilities is more reasonable, the professionalism reaches 100%, and the area utilization rate and comfort are higher than 85%. FACOPT algorithm and genetic algorithm are to compare the layout results. The results show that the method can obtain the optimal fitness value with a low number of iterations, and the shortest time is about 3.83 s.

In the future, the following issues should also be considered: exploring cheap and effective sensor materials to reduce the use cost of Internet of Things technology, coordinating government departments to transform gymnastics venues, planning and construction from a more reasonable point of view; dynamically analyzing the optimal layout of gymnastics venues, site selection should be considered more thoroughly, facility types should be adapted to local conditions, and the layout should be clear according to the user groups and scale; smart wearable devices should be used together with smart devices of monitoring facilities to unify data specifications; learn from advanced technology and experience to further optimize the model algorithm, reduce a large number of complex calculation steps in training and testing, reduce the amount of calculation, and improve the calculation speed.

## Figures and Tables

**Table 1:** Environment configuration.

Software or hardware conditions	Version or model
Microcontroller	ESP32-D0WDQ6, ATmega328P
Sensor	Sn3O4/RGO heterostructure, cotton/RGO/CN composites
Communication technology	5G
GIS technology	ArcGIS 10.5
WIMU	Wearable inertial measurement equipment
Software environment	Python 3.7.4
Aided design software	LabVIEW 2010

**Table 2:** Monitoring of gymnastics facilities.

Venue number	Temperature (degrees)	Wettability	Facility pressure	Gas mass	Physiological signal	Energy consumption
1	22.5	23	Normal	Good	Normal detection	General
2	21.8	35	Normal	Excellent	Normal detection	Larger
3	20.8	24	Normal	Good	Normal detection	General
4	23.1	36	Normal	Good	Normal detection	Smaller
5	22	30	Normal	Excellent	Normal detection	Smaller

## Data Availability

The experimental data used to support the findings of this study are available from the corresponding author upon request.
